# Supplementary figures and images for: Lack of bombesin receptor–activated protein attenuates bleomycin-induced pulmonary fibrosis in mice
Source: Life Sci Alliance. 2022 Jul 12;5(11):e202201368. doi: 10.26508/lsa.202201368 (PMC9275683; doi:10.26508/lsa.202201368)

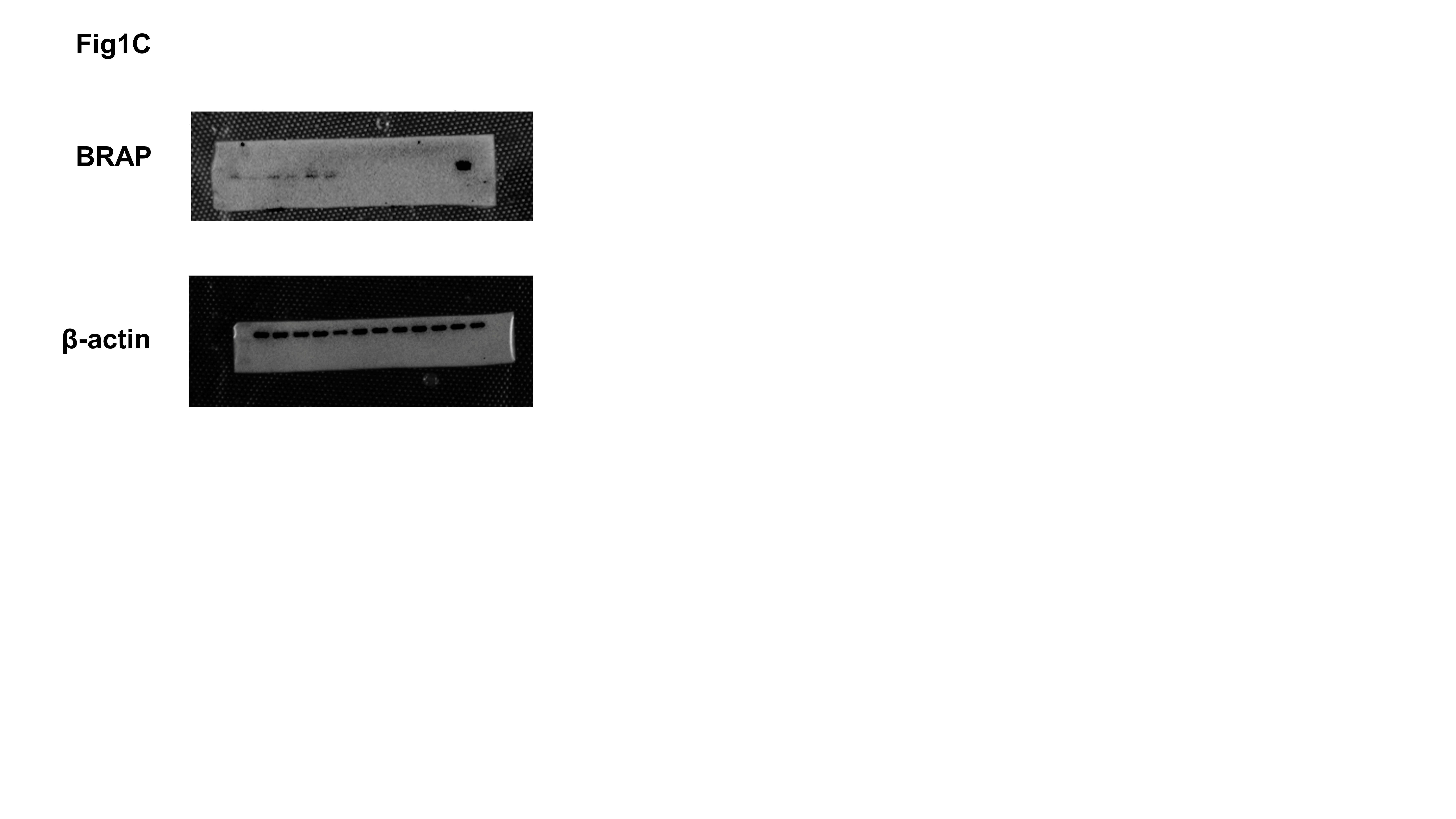

Supplement: Supplementary file 1 [file LSA-2022-01368_SdataF1.tif]

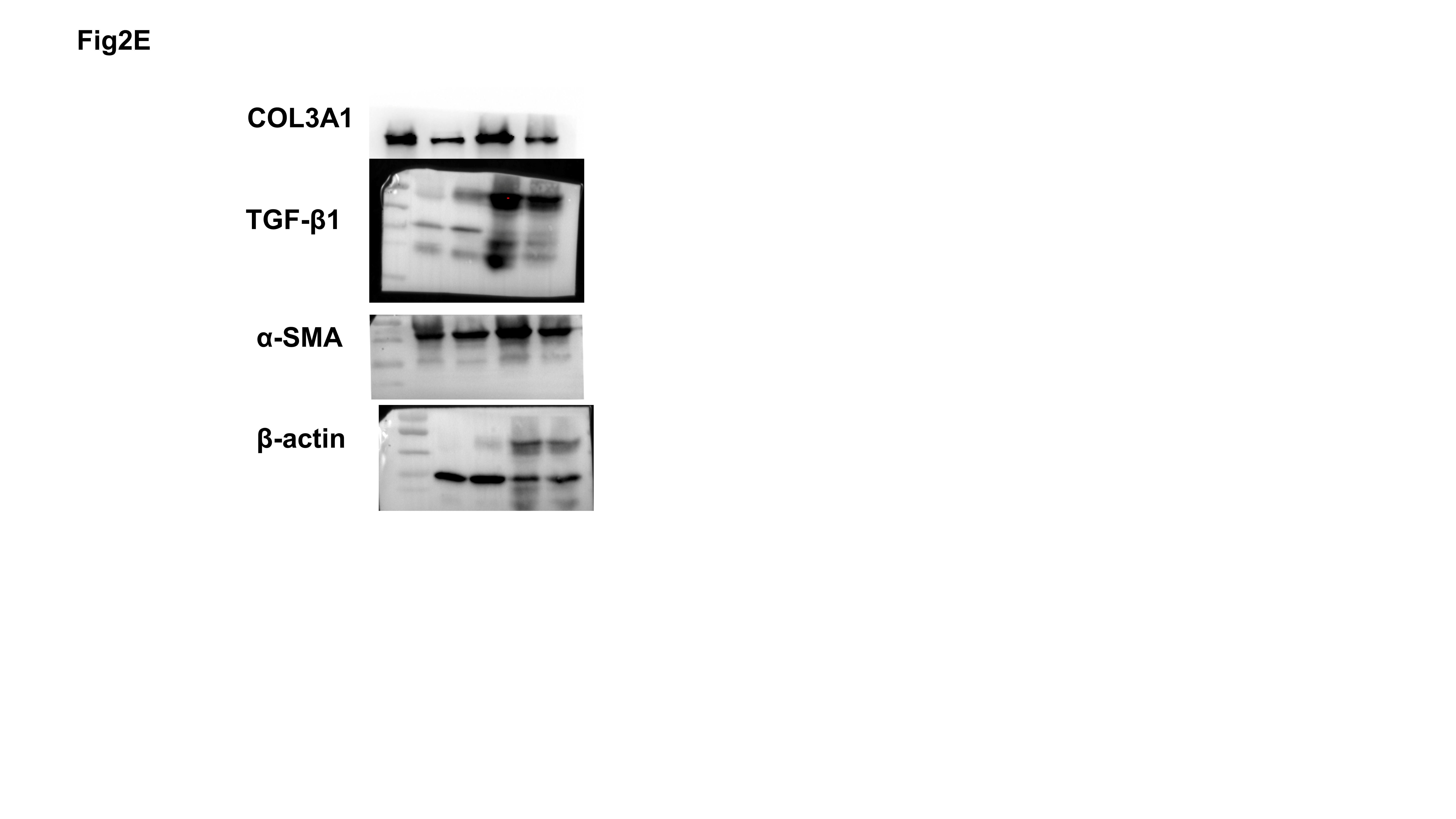

Supplement: Supplementary file 2 [file LSA-2022-01368_SdataF2.tif]

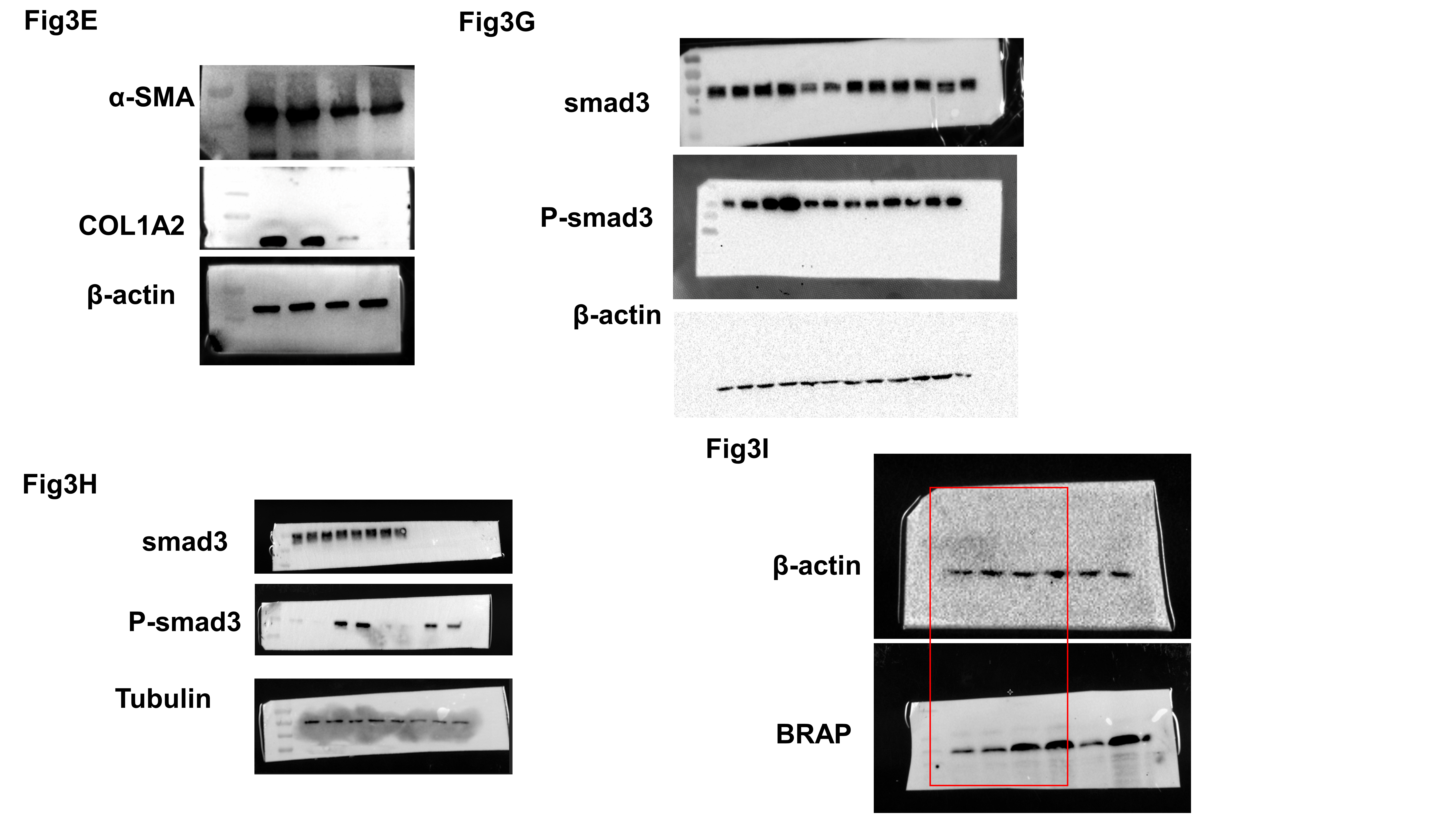

Supplement: Supplementary file 3 [file LSA-2022-01368_SdataF3.tif]

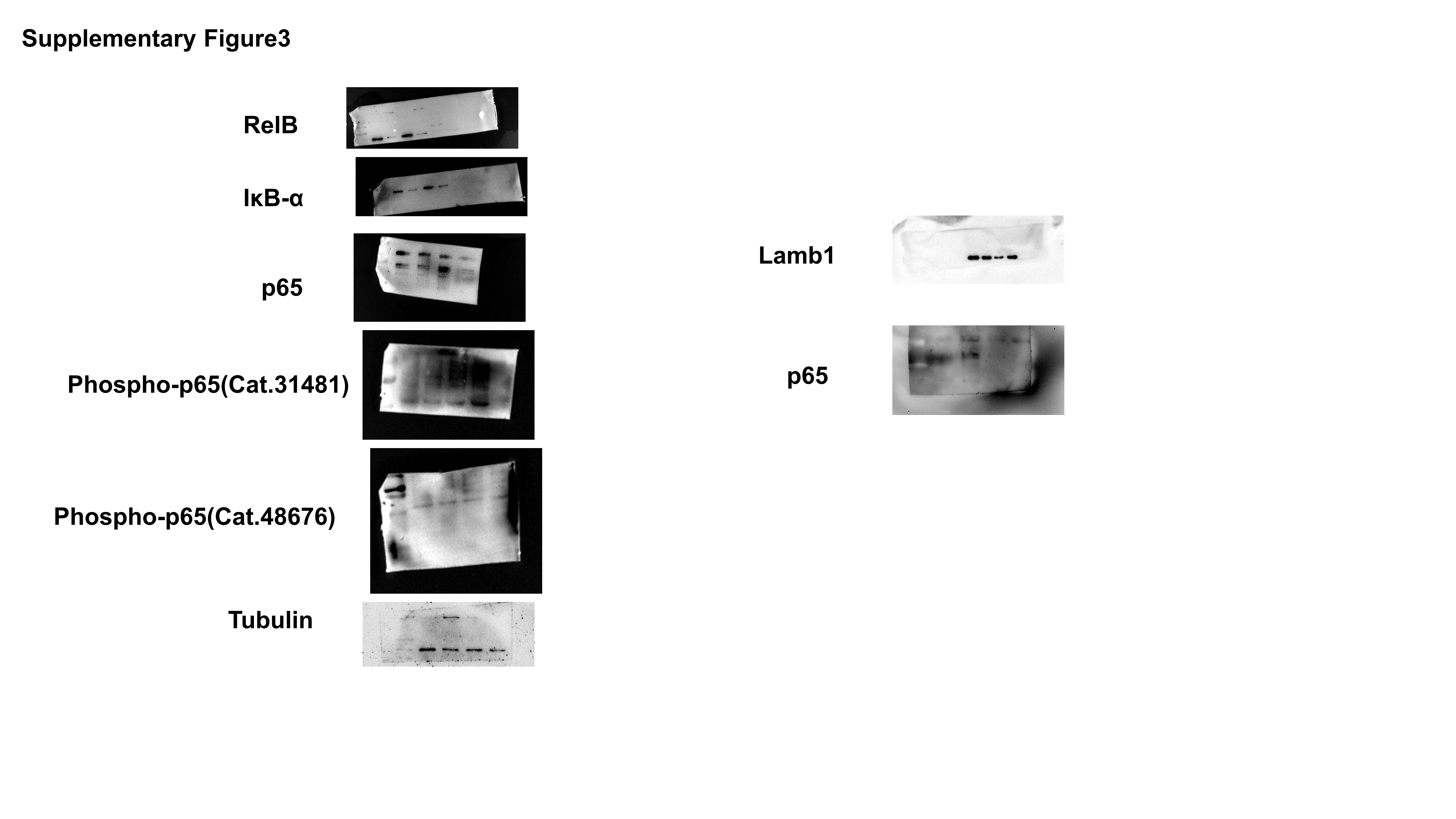

Supplement: Supplementary file 4 [file LSA-2022-01368_SdataFS3.tif]

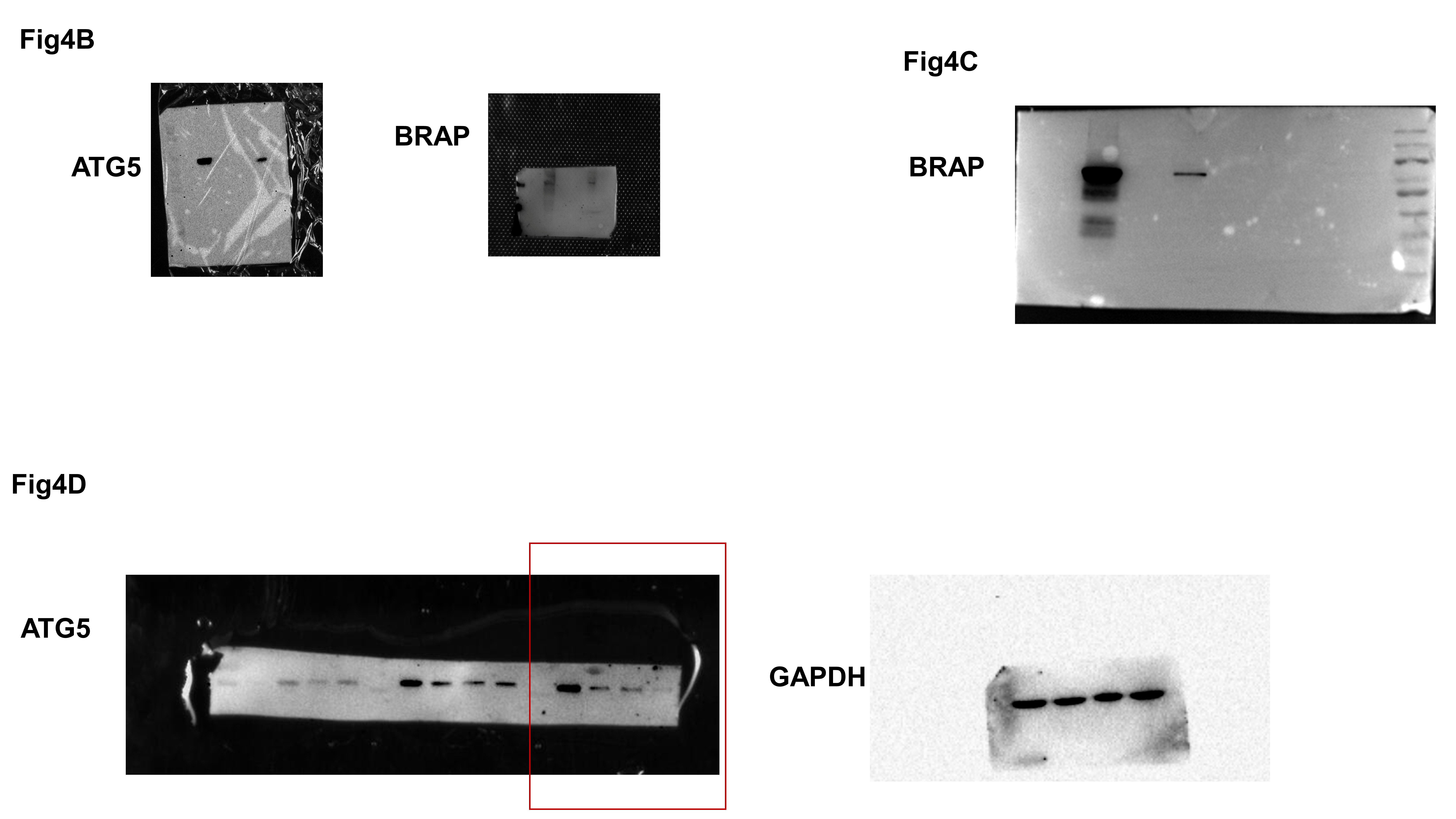

Supplement: Supplementary file 5 [file LSA-2022-01368_SdataF4.tif]

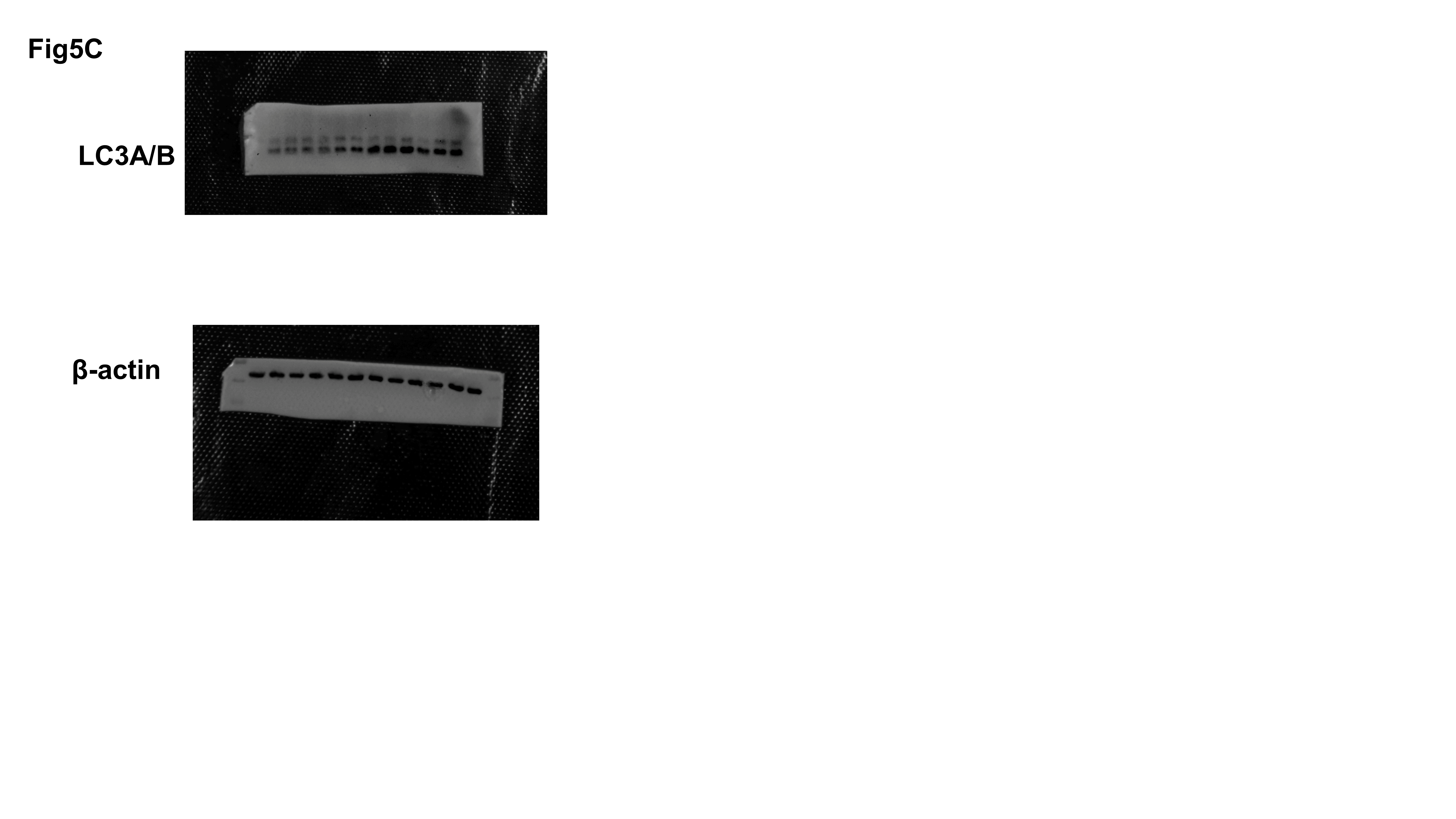

Supplement: Supplementary file 6 [file LSA-2022-01368_SdataF5.tif]

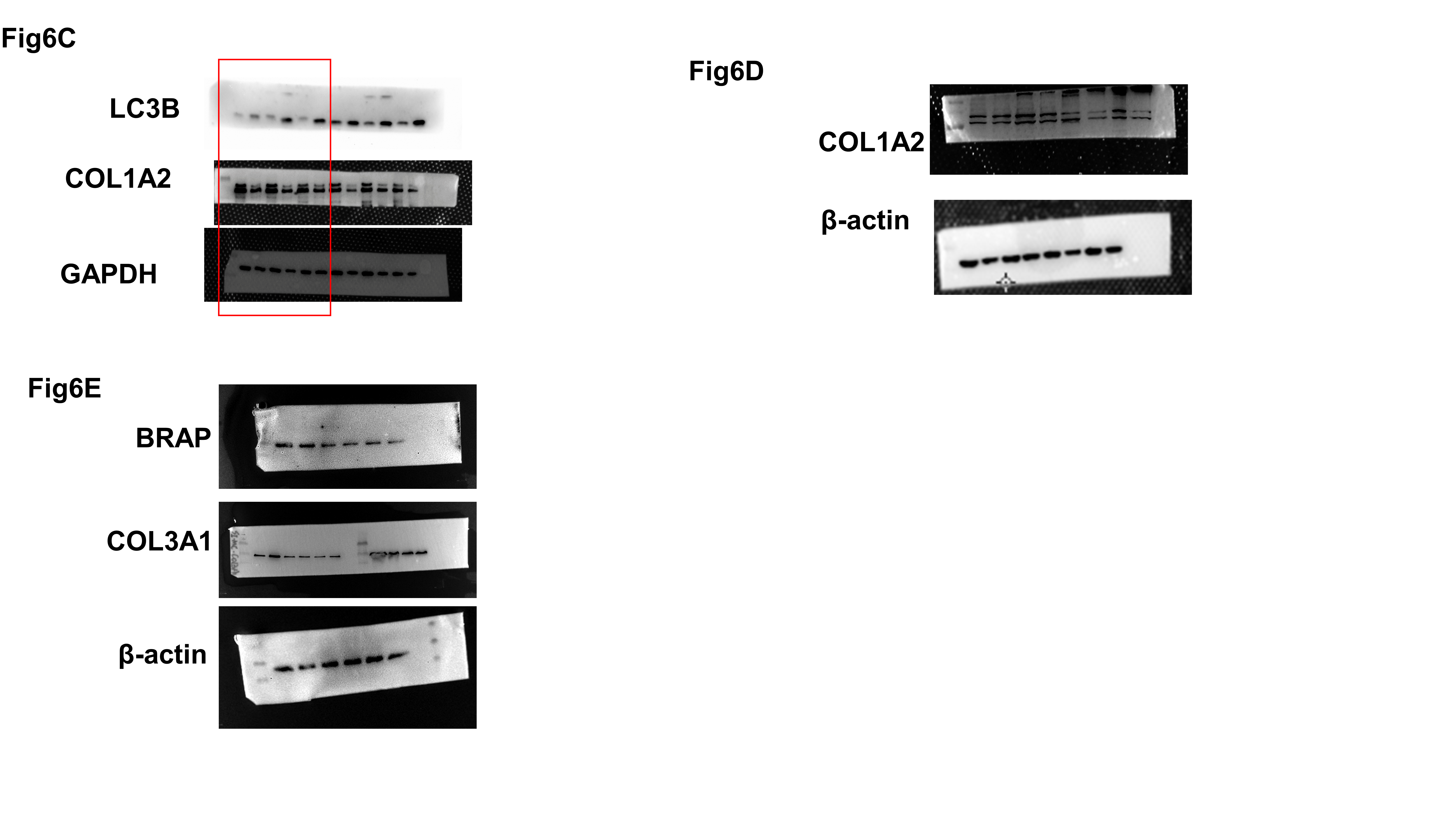

Supplement: Supplementary file 7 [file LSA-2022-01368_SdataF6.tif]
